# A Fully-Immersive Virtual Reality Setup to Study Gait Modulation

**DOI:** 10.3389/fnhum.2022.783452

**Published:** 2022-03-23

**Authors:** Chiara Palmisano, Peter Kullmann, Ibrahem Hanafi, Marta Verrecchia, Marc Erich Latoschik, Andrea Canessa, Martin Fischbach, Ioannis Ugo Isaias

**Affiliations:** ^1^Department of Neurology, University Hospital of Würzburg and Julius Maximilian University of Würzburg, Würzburg, Germany; ^2^Human-Computer Interaction, Julius Maximilian University of Würzburg, Würzburg, Germany; ^3^Department of Informatics, Bioengineering, Robotics and System Engineering, University of Genoa, Genova, Italy; ^4^Parkinson Institute Milan, ASST Pini-CTO, Milano, Italy

**Keywords:** gait modulation, virtual reality, obstacle avoidance, gait analysis, kinematics

## Abstract

**Objective**: Gait adaptation to environmental challenges is fundamental for independent and safe community ambulation. The possibility of precisely studying gait modulation using standardized protocols of gait analysis closely resembling everyday life scenarios is still an unmet need.

**Methods**: We have developed a fully-immersive virtual reality (VR) environment where subjects have to adjust their walking pattern to avoid collision with a virtual agent (VA) crossing their gait trajectory. We collected kinematic data of 12 healthy young subjects walking in real world (RW) and in the VR environment, both with (VR/A+) and without (VR/A-) the VA perturbation. The VR environment closely resembled the RW scenario of the gait laboratory. To ensure standardization of the obstacle presentation the starting time speed and trajectory of the VA were defined using the kinematics of the participant as detected online during each walking trial.

**Results**: We did not observe kinematic differences between walking in RW and VR/A-, suggesting that our VR environment *per se* might not induce significant changes in the locomotor pattern. When facing the VA all subjects consistently reduced stride length and velocity while increasing stride duration. Trunk inclination and mediolateral trajectory deviation also facilitated avoidance of the obstacle.

**Conclusions**: This proof-of-concept study shows that our VR/A+ paradigm effectively induced a timely gait modulation in a standardized immersive and realistic scenario. This protocol could be a powerful research tool to study gait modulation and its derangements in relation to aging and clinical conditions.

## Introduction

Bipedal walking is a remarkable ability of humans that requires highly complex neural control to effectively adapt in response to environmental challenges (Jahn et al., 2008; Queralt et al., 2008; Takakusaki, 2013; Tard et al., 2015; Corporaal et al., 2018; Nordin et al., 2019; Pozzi et al., 2019). Impairment of gait adaptation is common in older adults, and among the first indications of gait derangements in neurological diseases. This significantly increases the risk of falls (Caetano et al., 2016), resulting in fractures (Stalenhoef et al., 2002; World Health Organization, 2007), loss of independence (Tinetti et al., 1994; Stalenhoef et al., 2002; World Health Organization, 2007), poor quality of life, and high mortality (World Health Organization, 2007; Osoba et al., 2019).

Many studies have investigated overground gait adaptation in response to obstacles in healthy young and older adults (Sparrow and Tirosh, 2005; Weerdesteyn et al., 2018). However, precise measures of gait patterns in response to real world (RW) demands are scarce (Weerdesteyn et al., 2018), primarily due to the lack of setups in gait laboratories that can fully replicate everyday life environments (Sparrow and Tirosh, 2005).

Previous works used two main approaches to study gait modulation, with fixed (Vallis and McFadyen, 2005; Da Silva et al., 2011; Jansen et al., 2011; Yamada et al., 2011) or mobile obstacles (Gérin-Lajoie et al., 2005; Cinelli and Patla, 2007, 2008; Da Silva et al., 2011; Olivier et al., 2012, 2013; Basili et al., 2013; Huber et al., 2014; Knorr et al., 2016; Vassallo et al., 2017). Fixed obstacles have the advantage of easier standardization across trials and subjects, but they may induce anticipation and pre-planning (Yamada et al., 2011) and do not allow adequate study of the gait modulation that occurs in an outdoor environment, where moving obstacles are prevalent (Sparrow and Tirosh, 2005). With respect to fixed obstacles, moving obstacles cause larger changes in the gait pattern (Gérin-Lajoie et al., 2005), requiring higher mental processing costs (Cutting et al., 1995; Gérin-Lajoie et al., 2005) and being more challenging for people at high risk of falling (Osoba et al., 2020). Gait pattern changes include both gait trajectory (Gérin-Lajoie et al., 2005; Cinelli and Patla, 2007; Basili et al., 2013; Olivier et al., 2013; Vassallo et al., 2017) and velocity (Cinelli and Patla, 2008; Basili et al., 2013; Olivier et al., 2013; Huber et al., 2014; Knorr et al., 2016). In the presence of sufficient space, directional adjustments are preferred (Huber et al., 2014), but braking strategies (i.e., speed modulation) can also be present with obstacle crossing angles of 45° and 90° (Huber et al., 2014). Time constraints, including different obstacle velocities, can also affect gait adaptation. In fact, the (medio-lateral) safety margins for collision avoidance (Cinelli and Patla, 2007, 2008) and the step length (Da Silva et al., 2011) depend on the speed of the obstacle. These results highlight the importance of standardizing obstacle presentation to evoke similar kinematic responses across trials and subjects.

Some previous studies have used a person trained to walk with specific trajectories and speeds as the moving obstacle (Olivier et al., 2012, 2013; Basili et al., 2013; Huber et al., 2014; Knorr et al., 2016). This has the advantage of closely replicating an everyday situation but increases the variability in obstacle presentation, which could not be standardized in these studies. Other studies used robots (Vassallo et al., 2017), mannequins (Gérin-Lajoie et al., 2005; Cinelli and Patla, 2007, 2008), or remote-controlled objects (Da Silva et al., 2011) to improve the accuracy of obstacle presentation, but with some limitations. In particular, the movement of the obstacles was not dynamically adjusted to the behavior (trajectory or velocity) of the subject but fixed and arbitrarily chosen (Cinelli and Patla, 2007, 2008; Vassallo et al., 2017), based on normative data (Da Silva et al., 2011) or on the velocity of the subject during unperturbed walking (Gérin-Lajoie et al., 2005). In addition, in all but one study (Vassallo et al., 2017), the obstacle trajectory was fixed and did not adjust for the ongoing walking pattern of the subject.

Virtual reality (VR) holds great promise for overcoming many of these limitations. Experimental conditions in immersive VR are ecologically valid, realistic, highly controlled, and replicable in a safe environment (Bailenson et al., 2003). A VR setup allows accurate and real-time measurement of the position of the subject and the obstacle (Loomis et al., 1999; Bailenson et al., 2003) for standardization in its presentation. A VR setup can also be enriched with multiple cognitive and motor tasks (dual-task paradigm; Janeh et al., 2019) and perceptual loads (Martelli et al., 2019), requiring additional resources for planning and sensorimotor integration (Mirelman et al., 2011) that can aid a more comprehensive study of gait adaptation (Gérin-Lajoie et al., 2005; Konczak et al., 2009). Obstacle avoidance tasks in VR have shown great potential also for rehabilitation purposes in parkinsonian patients (Mirelman et al., 2011), post-stroke patients (Jaffe et al., 2004), and patients with cerebral palsy (Gagliardi et al., 2018). In most of these studies, however, the use of a treadmill limited the level of immersiveness, which can be resolved by implementing overground walking with a head-mounted display (HMD; Winter et al., 2021).

In recent years, several studies have been successful in developing VR paradigms capable of inducing gait modulation with virtual objects (Fajen et al., 2003; Fink et al., 2007; Gérin-Lajoie et al., 2008; Cirio et al., 2013; Argelaguet Sanz et al., 2015) or virtual persons (Argelaguet Sanz et al., 2015; Lynch et al., 2018; Olivier et al., 2018). Overall, these studies showed similar gait adaptation strategies in VR and RW, with the former characterized by higher obstacle clearance (Fink et al., 2007; Gérin-Lajoie et al., 2008; Argelaguet Sanz et al., 2015; Olivier et al., 2018) and slower velocity (Fink et al., 2007; Argelaguet Sanz et al., 2015). These differences may be due to uncertainties in obstacle localization, possibly caused by excessive attentional demands required by the VR environment (Gérin-Lajoie et al., 2008), absence of body rendering (Fink et al., 2007), and diminished field of view (Fink et al., 2007; Gérin-Lajoie et al., 2008). This latest hypothesis was, however, questioned by Jansen and coll., who showed kinematic gait changes during static obstacles avoidance only for a field of view as small as 40°×25° (Jansen et al., 2011), and by Knapp and Loomis, who found no underestimation of distances in relation to a decreased field of view (Knapp and Loomis, 2004).

All these studies have shown the great potential of VR in the study of gait modulation, but they are not without limitations. First, most of them used CAVE-like systems (Cruz-Neira et al., 1992) with joystick navigation, due to limited walking space (Lynch et al., 2018; Olivier et al., 2018). These devices are very expensive and require trained personnel, thus reducing their use in clinical and rehabilitation facilities. Second, studies of gait modulation in VR focused primarily on validating experimental setups previously used in RW rather than developing new ones. Static obstacles were preferred over moving obstacles, with the aim of understanding the impact of different characteristics of virtual obstacles on walking behavior (Bailenson et al., 2003; Argelaguet Sanz et al., 2015) or different avoidance strategies between VR and RW (Fajen et al., 2003; Fink et al., 2007; Gérin-Lajoie et al., 2008; Argelaguet Sanz et al., 2015). The few studies employing moving obstacles in VR (Lynch et al., 2018; Olivier et al., 2018) used joystick navigation and did not adjust the movement of the obstacle to the movement of the subject. The potential of VR in replicating everyday environments and standardizing the presentation of obstacles has yet to be fully exploited.

Ours is a proof-of-concept study that aimed to demonstrate the feasibility of using a fully-immersive VR environment to study overground gait adaptation and obstacle avoidance in a highly standardized manner. We tested this protocol on a small group of young healthy subjects and described biomechanical features of overground gait modulation for collision avoidance. We employed an HMD to ensure immersiveness and facilitate future clinical applications. A virtual agent (VA) was preferred over a virtual object to replicate one of the most common scenarios in daily life, which is walking while another pedestrian crosses the path (Basili et al., 2013; Olivier et al., 2013; Huber et al., 2014; Knorr et al., 2016). A full-bodied VA was shown to induce larger gait adaptation with respect to inanimate objects (Argelaguet Sanz et al., 2015; Lynch et al., 2018). For the first time, the movement of the object (i.e., the VA) was standardized based on the ongoing movement of the participant to ensure a constant perturbation across subjects and trials. The speed of the VA was defined so that participants were induced to modulate their gait to let the VA pass first. Indeed, when two pedestrians cross their paths, the one way contributes more to collision avoidance (both in terms of walking trajectory and speed changes) than the one passing first (Olivier et al., 2013; Knorr et al., 2016). This setup was designed for future studies on patients with Parkinson’s disease, where specific gait disturbances such as gait freezing predominantly occur during gait pattern modulation (e.g., confrontation with obstacles; Pozzi et al., 2019).

We had two main working assumptions: the first was that a highly realistic and immersive virtual environment would not alter the gait pattern. For this part of the study, our results should be considered preliminary, and we defer validation of our setup to future works with more subjects. The second hypothesis was that the presence of the VA would induce significant gait modulation, both in terms of stride velocity, length, and duration, and in terms of stride width, lateral trunk displacement, and lateral deviation of the gait trajectory. This second goal, especially for future clinical research applications, should be considered more relevant and the main purpose of this work.

## Methods

### Subjects

The absolute novelty of this study setup and the lack of preliminary results prevented us from performing an *a priori* power analysis to determine the sample size. For this proof-of-concept study, we studied a number of participants similar to previous studies of ground-based obstacle avoidance in VR (Bailenson et al., 2003; Fink et al., 2007; Gérin-Lajoie et al., 2008). We recruited 12 healthy young participants (seven males; age 23–40 years; Table 1). No participant suffered from any medical condition and was a professional athlete. All participants had a normal or corrected-to-normal vision. They had no previous experience with any VR device. The study was approved by the local Ethical Committee of the University of Würzburg (n. 103/20) and conformed to the declaration of Helsinki (World Medical Association, 2013). All subjects gave their written informed consent prior to participation.

**Table 1:** Demographic data and anthropometric measurements.

Gender (males/total (%))	7/12 (58.3)
Age (years)	29.3 (5.3)
Body height (cm)	168.9 (8.2)
Foot length (cm)	24.4 (1.4)
Limb length (cm)	90.2 (4.5)
Weight (kg)	66.3 (10.6)
BMI (kg/m^2^)	23.3 (3.9)

*Values are shown as mean (standard deviation) unless otherwise indicated. Abbreviations: BMI, body mass index*.

### Study Protocol

**Figure 1:**
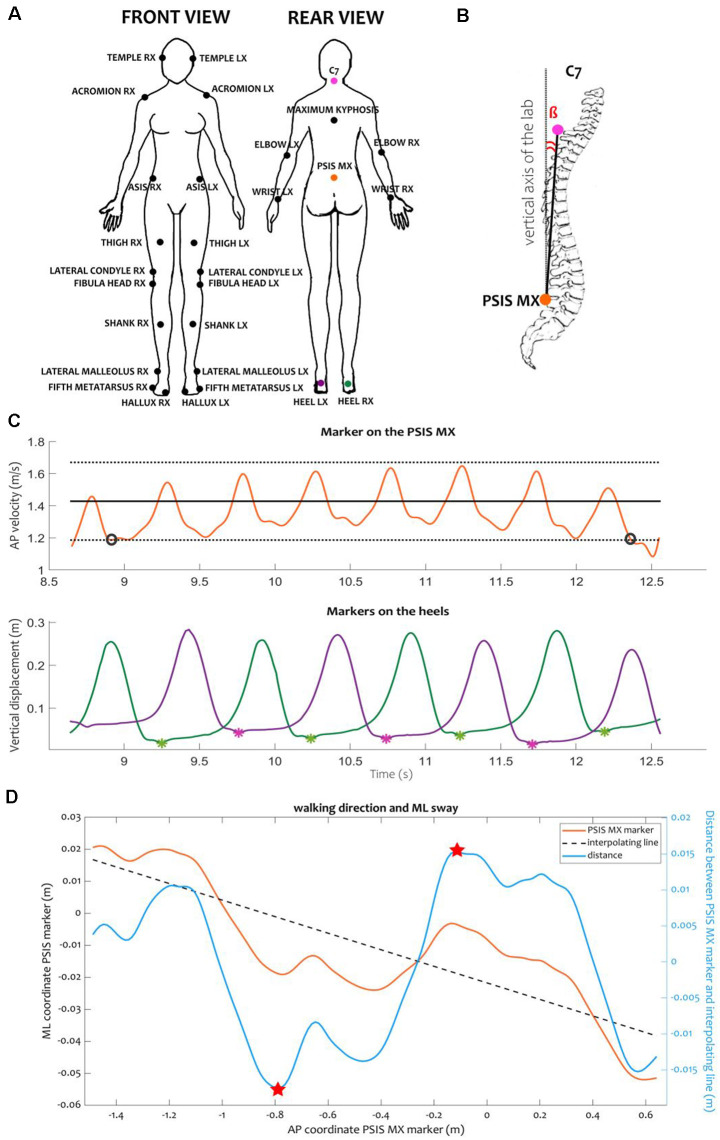
Kinematic protocol and variables. **(A)** Position of the markers according to the LAMB protocol (Palmisano et al., 2019). Colored markers were used for the computation of kinematic events and variables. **(B)** Representation of the trunk inclination. Trunk inclination β was defined as the angle between the vertical axis of the laboratory and the vector connecting the markers placed on the middle point between the PSIS (PSIS_MX) and the C7 vertebra. **(C)** Example of computation of steady-state velocity and identification of heel contacts for one RW trial. We defined the steady-state velocity specific for each subject as the average (black solid line) ± the standard deviation (black dotted lines) of the AP velocity of the PSIS_MX marker computed in the central portion of the calibration volume. Only the interval during which the velocity was consistently inside this range (between the black circles) was considered for computing the gait cycle parameters. Inside the window at steady-state velocity, we identified the heel contacts as the local minima (asterisks) of the vertical tracks of the markers placed on the heels (green and purple lines for the right and left heels, respectively). **(D)** Example of ML sway and walking direction during a RW trial. We computed the ML sway as the range of the distance (light blue line) between the trajectory of the PSIS_MX marker in the transversal plane (orange line) and its interpolating line (black dashed line). The range was computed as the difference between the maximum and minimum values of the distance (indicated here as red stars). The direction of the walking trajectory was computed as the angular coefficient of the linear regression line interpolating the PSIS_MX trajectory in the transversal plane. Abbreviations: AP, anterior-posterior; C7, seventh cervical vertebra; ML, medio-lateral; PSIS, posterior-superior iliac spines.

The study protocol consisted of four sessions, each comprising 20 walking trials on a 10 m walkway. Kinematics were recorded using an optoelectronic system with six cameras (sampling rate 100 Hz, SMART DX-400, BTS Bioengineering, Italy) and a set of 29 markers placed on anatomical landmarks (Figure 1A; Palmisano et al., 2019, 2020a, b; Farinelli et al., 2020). During the first session, the subjects walked back and forth on the walkway in the RW. In the second session, the subjects walked in the same fashion, but in the VR environment (VR/A-). The last two sessions were performed in the same VR environment, with the addition of a VA (VR/A+). A verbal “start” and “stop” signal defined the beginning and end of the session. Between sessions, subjects were allowed to rest. Before starting the recording, subjects performed three-five walking trials in VR to become acquainted with the environment. In all conditions, participants were asked to walk at their natural (preferred) speed. In VR/A+, participants were informed that the VA would cross their path once in each walking trial and instructed to adapt their gait to avoid collision with the VA without stepping off the walkway. Sessions were presented in the same order for all recruited subjects (i.e., RW, VR/A-, VR/A+). Synchronization of acquiring devices was achieved using a transistor-transistor-logic (TTL) signal recorded at the same time by the VR and the SMART systems.

### Virtual Laboratory Environment

**Figure 2:**
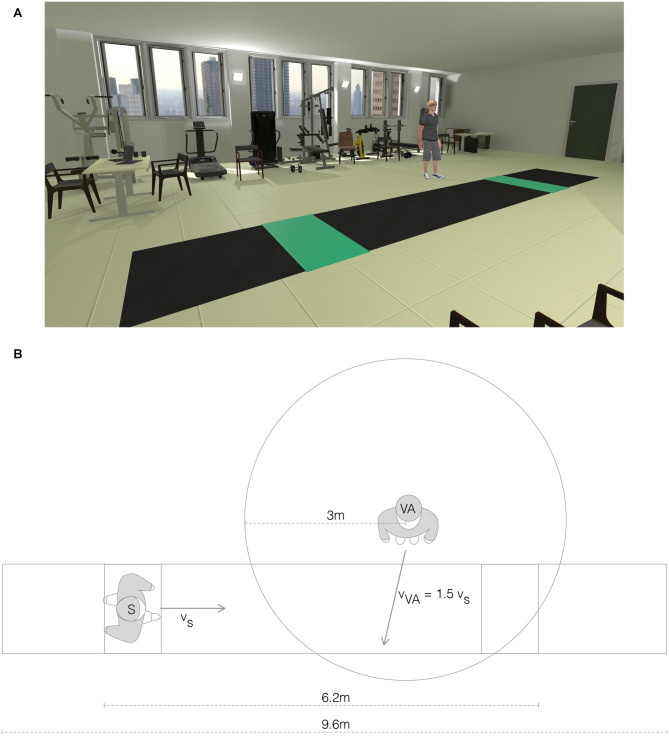
Virtual reality environment. **(A)** View of the virtual reality (VR) environment with the virtual agent (VR/A+ condition). **(B)** Top view schema of the VR environment representing the relative positions of the subject (S) and the virtual agent (VA).

The VR environment was made with Unity (Unity Technologies, USA). It was displayed to the subjects *via* a wireless HMD (Vive Pro, HTC, USA) connected to a PC (Intel Core i9-10900X 10 cores, NVIDIA GEFORCE 11 GB RTX 2080, 32 GB RAM). A virtual laboratory environment was created using one-to-one mapping to closely resemble the real laboratory. We did not apply any translational gains (Williams et al., 2006) or even redirected walking techniques (Steinicke et al., 2008), as they showed a detrimental effect on the gait pattern and altered the behavior of the subject during walking (i.e., subjects had the tendency to look down toward the floor during walking; Janeh et al., 2017). We positioned the virtual world so that the virtual walkway was aligned with the real one. In the virtual laboratory, two green tiles were visible at both ends of the walkway (Figure 2A). Participants had to repeatedly walk back and forth from one green tile to the other. At the beginning of each trial, the subject could see the VA standing 5 m in front and 1.5 m to the side (left and right alternately) of the green tile from which the subject was starting. The arrival of the subject on the green tile, before turning around, determined the repositioning of the VA for the next walking trial (Figure 2A). The VA was programmed to cross the walking path of the subject in a standardized fashion. Specifically, the VA started walking in a straight line towards the subject’s pathway when the subject-to-agent distance was 3 m, with a constant speed equal to 1.5 times the speed of the subject at the instant of the VA start (Figure 2B). The trajectory of the VA was set to cross the walking pathway of the subject at 1 m distance from the subject, assuming that no gait adaptation took place. To quantify sickness elicited by our VR setup, we used the Simulator Sickness Questionnaire (SSQ; Kennedy et al., 1993).

### Data Analysis

Kinematic data were extracted using *ad hoc* Matlab algorithms. For the RW and VR/A- sessions, we analyzed only the strides at steady-state velocity. A stride was defined as the interval between two subsequent heel contacts of the same foot, detected as local minima in the vertical displacement of the markers placed on the heels (Figure 1C). Steady-state velocity was defined as the mean ± standard deviation of the anterior-posterior velocity of the marker placed on the middle point between the posterior superior iliac spines [(PSIS_MX), approximating the center of mass (Yang and Pai, 2014)], computed in the central portion of the calibration volume (Figure 1C). For the VR/A+ trials, we identified a gait modulation phase as the time between the movement onset of the VA and the instant when the subjects regained their steady-state velocity, as identified in the VR/A- session. In the modulation phase, we identified three strides: first, second, and third modulator. For each stride (for RW and VR/A-) or modulator (for VR/A+), we measured the spatiotemporal parameters (i.e., stride length, width, duration, and velocity) and the trunk inclination as the angle between the vertical axis of the laboratory and the vector from the PSIS_MX marker to the marker on the seventh cervical vertebrae (C7; Figure 1B). For steady-state velocity walking (in RW and VR/A-) and for the gait modulation phase (in VR/A+), we measured the walking direction as the angular coefficient of the linear regression line interpolating the PSIS_MX trajectory in the transversal plane (Figure 1D). We also estimated the mediolateral sway as the range of the distance between the points of the PSIS_MX marker and the regression line in the transversal plane (Figure 1D).

### Statistical Analysis

All variables were averaged for each subject across trials, and one value represented the subject in each condition. We used the Friedman and Wilcoxon matched-pairs tests to investigate differences between the RW, VR/A-, and VR/A+ conditions. A *p*-value of 0.05 corrected with the Bonferroni method was used as a threshold for statistical significance for both the Friedman and the *post-hoc* tests.

## Results

Demographic features and anthropometric measures are summarized in Table 1. None of the participants reported any discomfort or symptoms due to the VR during or after the study (SSQ total score <5).

No statistically significant differences were observed between the RW and VR/A- conditions for any parameter (Table 2).

**Table 2:** Kinematic measures.

Condition	RW	VR/A-	VR/A+		
			M1	M2	M3
Stride length (cm)	132.4 (9.2)^a,b^	129.0 (11.2)^d,e^	96.1 (16.6)^a,d,g,h^	111.2 (13.0)^b, e, g, i^	124.0 (10.1)^h,i^
Stride width (cm)	8.2 (3.2)^a^	8.1 (2.3)^d^	10.3 (2.2)^a,d,h^	8.9 (2.9)	8.2 (2.2)^h^
Stride duration (s)	1.1 (0.1)^a,b,c^	1.1 (0.1)^d,e,f^	1.3 (0.2)^a,d^	1.3 (0.2)^b,e^	1.2 (0.1)^c, f^
Stride velocity (cm/s)	122.8 (13.2)^a, b, c^	117.5 (16.1)^d, e, f^	78.4 (15.7)^a,d,g,h^	88.1 (17.4)^b,e,g,i^	103.2 (13.4)^c,f,h,i^
Trunk inclination (°)	4.7 (1.6)^c^	4.2 (1.4)^e,f^	4.8 (1.9)^g^	5.6 (1.7)^e,g^	6.1 (1.7)^c,f^

*Values are shown as mean (standard deviation). The letters “a, b, c, d, e, f, g, h, i” represent significant difference between conditions (Wilcoxon matched-pairs test for pairwise comparisons with Bonferroni correction for multiple comparisons). Abbreviations: RW, real world; VR/A-, virtual reality without virtual agent; VR/A+, virtual reality with virtual agent (i.e., perturbed gait); M1, first modulator; M2, second modulator; M3, third modulator*.

**Figure 3:**
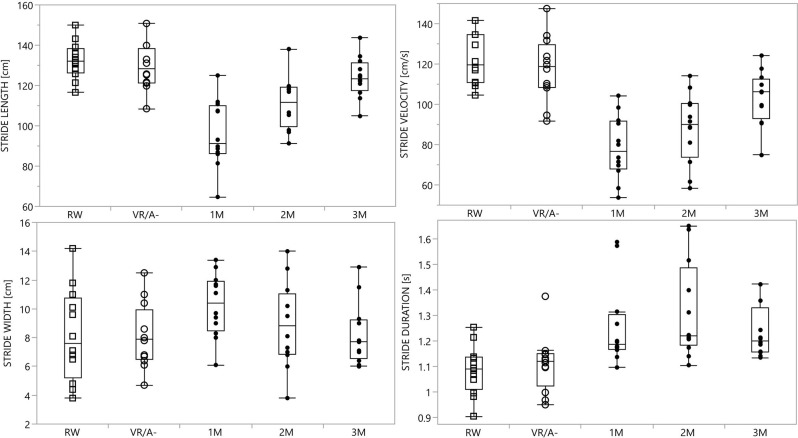
Kinematic measurements of the stride. Graphical representation of kinematic measurements of the stride in all conditions; please see Table 2 for kinematic values and statistics. Data are shown as the mean and standard error of the mean. Abbreviations: RW, real world; VR/A-, virtual reality environment without virtual agent; M1, first modulator; M2, second modulator; M3, third modulator.

We showed a clear gait pattern modulation during walking in the VR/A+ condition. Stride length and velocity decreased in all modulators, being lowest at the first modulator and increasing progressively from the first to the third modulator. The stride width selectively increased at the first modulator. All modulators had a longer duration than RW and VR/A- strides (Table 2, Figure 3). Trunk inclination increased during all modulators and peaked significantly at the third modulator (Table 2). The walking direction and mediolateral sway also increased during VA avoidance with respect to both control conditions (Table 3).

**Table 3:** Walking direction and mediolateral sway.

Measure	RW	VR/A-	VR/A+
Walking direction (°)	1.1 (0.3)^§^	1.4 (0.5)*	3.3 (1.0)*^§^
Mediolateral sway (cm)	5.4 (0.8)^§^	5.3 (1.0)*	7.9 (1.6)*^§^

Values are shown as mean (standard deviation). *, ^§^ represent significant differences between conditions (Wilcoxon matched-pairs test for pairwise comparisons with Bonferroni correction for multiple comparisons). Abbreviations: RW, real world; VR/A-, virtual reality without virtual agent; VR/A+, virtual reality with virtual agent (i.e., perturbed gait).

## Discussion

Our study showed that a fully-immersive VR environment is an effective setup to induce gait adaptation for obstacle avoidance. The consistent and replicable gait modulation induced by the VA in all participants indicates that this is a promising tool to study gait adaptation in a safe, highly-standardized, controlled, and lifelike environment.

The proposed VR environment did not induce changes *per se* in the basic kinematic features of gait. Still, we cannot rule out that the limited sample size may have prevented capturing significant differences, especially considering that previous studies described some alterations (e.g., stride length and velocity, cadence, heading angle) between walking in real and virtual environments (Hollman et al., 2006; Menegoni et al., 2009; Katsavelis et al., 2010; Janeh et al., 2017). Future studies are warranted to confirm these results in larger case series.

In all subjects, interaction with the VA induced significant changes in both gait trajectory (Table 3) and velocity, particularly the latter (Table 2 and Figure 3). This was expected, based on the crossing angle of the VA (Huber et al., 2014) and the presence of the walkway (Figure 2), and supports previous observations on the role of speed adjustments in obstacle avoidance (Cinelli and Patla, 2008; Basili et al., 2013; Olivier et al., 2013; Huber et al., 2014; Knorr et al., 2016). By defining a limited space for gait modulation, we made speed changes alone insufficient to avoid a collision with the VA (Huber et al., 2014), thus requiring parallel adjustments in gait trajectory (Huber et al., 2014) and step length (Gérin-Lajoie et al., 2005). The recovery of stride length, only partially accompanied by an increase in stride velocity, made the second modulator the longest in duration (Figure 3). Of note, changes between modulators were smooth, and values gradually restored to the unperturbed range during the second and third modulators (Figure 3).

Our VR paradigm also induced some additional mediolateral changes, which consisted of an increase in stride width and medio-lateral sway (Tables s 2 and 3; Gérin-Lajoie et al., 2005; Cinelli and Patla, 2007, 2008; Huber et al., 2014). The increase in stride width may reflect a strategy to ensure balance for the avoidance of the VA, which perturbs postural stability as suggested by the increased medio-lateral sway in the VA/A+ condition (Table 3). Changes in stride width, however, were inconsistent across subjects and these findings should be further confirmed in larger cohorts.

Finally, we noticed an increase in trunk inclination during the second and especially third modulator. This could be an attempt to maintain sufficient personal space relative to the VA (Gérin-Lajoie et al., 2005, 2006, 2008; Argelaguet Sanz et al., 2015; Lynch et al., 2018; Olivier et al., 2018), particularly during strides in which the VA was close to the participant (i.e., the second and third modulators).

One limitation of our study is the choice not to randomize between conditions (i.e., RW, VR/A, and VR/A+). The main reasons for this are that switching repeatedly from RW to VR can induce discomfort (e.g., dizziness and nausea), and requires additional time to remove and reposition the HMD, reducing subject compliance and the number of overall trials. Randomization between the VR/A- and VR/A+ conditions would have resulted in wait-and-see behavior, with additional gait changes given just by the expectation of whether the VA would begin moving. Instead, we wanted subjects to know that they needed to modulate their gait.

In conclusion, our VR setup was able to effectively induce timely gait modulation in a standardized, immersive, and realistic scenario that simulated a person crossing the path of the participant. Modulation involved both temporal and spatial adaptations of the gait cycle, as well as gait trajectory and trunk inclination. The use of this protocol in older subjects and patients with gait disorders could be useful to elucidate specific alterations in gait adaptation, and have diagnostic and therapeutic (physical therapy) value for future studies (Dockx et al., 2016; McCrum et al., 2017). In particular, we envision that the adaptive gait behavior induced by our VR paradigm may represent an ideal trigger for the occurrence of gait freezing episodes in patients with Parkinson’s disease and other neurological disorders (Fasano et al., 2017; Pozzi et al., 2019). This assumption is based on our experience and previous studies describing the occurrence of gait freezing episodes, mainly during modulation of gait when facing an obstacle under conditions of temporal or spatial constraint (Nieuwboer et al., 2001; Hausdorff et al., 2003; Pozzi et al., 2019).

## Data Availability Statement

The raw data supporting the conclusions of this article will be made available by the authors, without undue reservation.

## Ethics Statement

The study involved human participants. It was approved by the local Ethical Committee of the University of Würzburg (no. 103/20) and conformed to the declaration of Helsinki. All subjects gave their written informed consent prior to participation.

## Author Contributions

CP: conceptualization, methodology, software, formal analysis, investigation, data curation, writing—original draft, and funding acquisition. PK: conceptualization, methodology, software, writing—original draft. IH: investigation, formal analysis, writing—original draft. MV: investigation, writing—review and editing. ML: conceptualization, methodology, writing—review and editing. AC: conceptualization and methodology. MF: conceptualization, methodology, resources, writing—review and editing, and supervision. IUI: conceptualization, methodology, formal analysis, investigation, resources, writing—review and editing, supervision, project administration, and funding acquisition. All authors contributed to the article and approved the submitted version.

## Conflict of Interest

The authors declare that the research was conducted in the absence of any commercial or financial relationships that could be construed as a potential conflict of interest.

## Publisher’s Note

All claims expressed in this article are solely those of the authors and do not necessarily represent those of their affiliated organizations, or those of the publisher, the editors and the reviewers. Any product that may be evaluated in this article, or claim that may be made by its manufacturer, is not guaranteed or endorsed by the publisher.
